# Increased Substitution Rates Surrounding Low-Complexity Regions within Primate Proteins

**DOI:** 10.1093/gbe/evu042

**Published:** 2014-02-25

**Authors:** Carolyn Lenz, Wilfried Haerty, G. Brian Golding

**Affiliations:** ^1^Department of Biology, McMaster University, Hamilton, Ontario, Canada; ^2^MRC Functional Genomics Unit, Department of Physiology, Anatomy and Genetics, Henry Wellcome Building of Gene Function, University of Oxford, United Kingdom

**Keywords:** low-complexity region, substitution, primate, mutation

## Abstract

Previous studies have found that DNA-flanking low-complexity regions (LCRs) have an increased substitution rate. Here, the substitution rate was confirmed to increase in the vicinity of LCRs in several primate species, including humans. This effect was also found among human sequences from the 1000 Genomes Project. A strong correlation was found between average substitution rate per site and distance from the LCR, as well as the proportion of genes with gaps in the alignment at each site and distance from the LCR. Along with substitution rates, *d*_N_/*d*_S_ ratios were also determined for each site, and the proportion of sites undergoing negative selection was found to have a negative relationship with distance from the LCR.

## Introduction

More than half of the human genome is composed of repetitive sequences (transposable elements, micro- and minisatellites) often defined as “junk” DNA, and even the 1.2% of our genome that includes all the protein coding genes is not free of these repetitive sequences. In humans, ∼14% of all proteins include at least one peptide sequence characterized by a low information content as a consequence of the repetition of a handful of amino acids (Golding 1999; Marcotte et al. 1999; Huntley and Golding 2000, 2002; Haerty and Golding 2010). The archetype of these peptide sequences is the reiteration of a single residue encoded by a trinucleotide repeat whose motif can be expanded up to 265 times in some severe cases of Huntington’s disease (Milunsky et al. 2003). Additionally, low-complexity amino acid sequences, including single amino acid repeats, are significantly enriched within specific classes of proteins such as transcription factors and proteins involved in development (Alba and Guigo 2004; Faux et al. 2005, 2007; Huntley and Clark 2007).

Like their noncoding counterparts, trinucleotide repeats within protein coding sequences, and more generally sequences encoding low-complexity regions (LCRs), are believed to evolve neutrally because of their low nucleotide conservation (Schmid and Tautz 1999; Huntley and Golding 2000; Alba and Guigo 2004; Huntley and Clark 2007) and their propensity to rapidly vary in size because of replication slippage (Ellegren 2004) and unequal recombination (Zilversmit et al. 2010). As a consequence of these mutational processes, the propensity of both noncoding and coding repeats to vary in size is directly correlated to their length homogeneity (Alba et al. 1999, 2001). Additionally, these sequences are found to be enriched in regions of proteins that are often associated with relaxed purifying selection such as alternatively spliced exons, interdomain sequences, regions far from functional domains, or at the surface of the protein in contact with the solvent (Romero et al. 2001; Haerty and Golding 2010).

In the case of noncoding microsatellites, mutation rates have been estimated to be 100–10,000 times greater than for other point mutations (Ellegren 2004). Although the vast majority of these sequences are often considered to evolve neutrally and their high mutability does not affect their host’s fitness, the evolution of many coding repetitive sequences greatly departs from neutral expectations, and evidence of selection acting on many of these sequences have been reported in a wide range of species (see Haerty and Golding 2010 for review).

Until recently, all analyses focused on describing the mutability of the repeats and their evolutionary patterns (Huntley and Golding 2002; Alba and Guigo 2004; Faux et al. 2005; Haerty and Golding 2010; Mularoni et al. 2010). However, the instability of a repeat is also likely to correlate with nucleotide variation within its flanking nucleotide sequences, either as a manifestation of a local relaxation of selection or as a direct consequence of a mutagenic effect of the repeat. For instance in *Escherichia coli*, instability at a trinucleotide repeat was associated with increased variation at adjacent repeats (Blackwood et al. 2010). Furthermore, multiple recent studies showed that insertion/deletions (indels) tend to be associated with an increased density of polymorphic sites within their flanking regions, suggesting that the high mutation rate and the size instability of low-complexity sequences in the genome also affects the sequences surrounding these regions (Vowles and Amos 2004; Huntley and Clark 2007; Amos et al. 2008). This observation of increased nucleotide variation near indels has been extended to microsatellite sequences (Siddle et al. 2011; McDonald et al. 2011; Amos 2013) and sequences encoding LCRs within proteins in *Drosophila* (Huntley and Clark 2007) and *Plasmodium falciparum* (Haerty and Golding 2011).

These recent findings extend the mechanisms proposed to explain the observed increased nucleotide variation near indel sequences (McDonald et al. 2011). Despite a study in *Drosophila melanogaster*, which ascribed the elevated density of single nucleotide polymorphism (SNPs) in the vicinity of indels to population admixture (Massouras et al. 2012), most analyses thus far have found an association between mutagenic effects with indels and repeated sequences. The increased mutation rate was first associated to heterozygous indels leading to error during DNA repair (Tian et al. 2008). In contrast McDonald et al. (2011) attributed the observed increased mutation rate to the recruitment of error-prone DNA polymerases at stalled replication forks due to repetitive sequences (but see Jovelin and Cutter 2013 for review). Finally, the observation of an increased transversion rate within the flanking regions of indels tend to favor this latter hypothesis (McDonald et al. 2011; Jovelin and Cutter 2013).

In light of the high number of protein coding sequences that contain these rapidly evolving low-complexity repeats within eukaryote genomes and their potential mutagenic effect within their surrounding sequences, one can wonder about the extent of such instability, its impact on the coding sequence, and the intensity of selective pressures, if any, exercised on these regions. Because the most deleterious mutations are expected to be ephemeral (Fu et al. 2013; Kiezun et al. 2013), in order to accurately quantify the mutational effect of the repetitive sequences and the selection acting on variation surrounding these regions, the analysis needs to focus on the comparison of closely related species or individuals within the same species. Most studies so far have been limited to a small number of genomes; however, the recent release of fully sequenced human genomes as part of the 1000 Genomes initiative (1000 Genomes Project Consortium 2010) gives us unprecedented power to assess the mutagenic effect of repetitive sequences within human populations and to quantify the strength of selection acting on these genomic regions.

Here we characterize the substitution pattern within primate proteins surrounding these LCRs. We also examine the nucleotide polymorphism in the vicinity of these repeated sequences within human populations. We show that LCRs significantly affect the evolution of their surrounding sequences, leading to both increased substitution and mutation rates within protein-coding sequences. With the availability of abundant data, we found an extremely strong relationship between the distance from the nearest LCR and the number of substitutions and indels seen in protein-coding DNA. Finally, a strong evidence of negative selection implies that these substitutions are occurring in functional, conserved sequences.

## Materials and Methods

Representative *Homo sapiens* (human, GRCh37), *Pan troglodytes* (chimpanzee, CHIMP2.1.3), *Gorilla gorilla* (gorilla, gorGor2), *Pongo pygmaeus* (orangutan, PPYG2), and *Macaca mulatta* (rhesus macaque, MMUL_1) genomes were downloaded from Ensembl release 62 (Flicek et al. 2011). The protein sequences were searched for regions of low-complexity with SEG (Wootton and Federhen 1993), using a window size of 15 bp and a complexity threshold of 1.9. Using this method, 7,387 LCRs were identified in humans. LCRs from the four other primate species were found and compared with find orthologs based on location within the protein as well as identity between the sequences, and we identified 5,584 LCRs (in 2,425 genes) shared by all five species (table 1). Data from the 1000 Genomes Project (1000 Genomes Project Consortium 2010) were downloaded and LCRs that corresponded to the LCRs associated with the primate flanking regions were identified. The translated protein sequences containing these LCRs were aligned using Muscle (Edgar 2004).

**Table 1:** Number of 1-to-1 Orthologous LCRs Shared between Each Pair of Species

	Human	Chimpanzee	Gorilla	Orangutan	Macaque
Human	7,387	6,908	6,776	6,635	6,662
Chimpanzee	—	7,208	6,635	6,611	6,565
Gorilla	—	—	7,098	6,461	6,412
Orangutan	—	—	—	6,957	6,414
Macaque	—	—	—	—	6,454

The SNP calls, including the allele frequencies, for 178 individuals of African origin (1000 Genomes Project Consortium 2010), were obtained, and the distance from the nearest LCR was found for all SNPs. Using the pairwise genome alignments downloaded from the UCSC database, the nucleotides were compared with homologous sites in macaque and chimpanzee to determine the ancestral state of the SNPs using maximum parsimony. Because we expect the most recent SNPs to have low allelic frequency, we assessed the relationship between the allelic frequency of each SNP and its distance to the LCR to assess the mutagenic propensity of LCR-encoding sequences on their flanking sequences and their potential response to selection. Only SNPs from protein-coding regions were used, because the more relaxed selection outside of coding regions could alter any relationship. Although a lower SNP frequency could also indicate more deleterious alleles, unless the presence of LCR was increasing the substitution rate or causing a relaxation in selection, we do not expect a distributional bias. Distance to the nearest protein-coding LCR, including introns and intergenic regions, was calculated. The various functional categories of the protein products of the genes were downloaded from Gene Ontology (GO) (Gene Ontology Consortium 2000) to see if the genes studied tended to be associated with any particular functional category.

The protein-coding regions containing LCRs were scrutinized to determine whether they had “suitable” flanking regions. To get a clear picture of how distance from an LCR-encoding sequence affects substitution rate, we identified flanking regions that did not overlap with those of another LCR and were not interrupted by either a transcription start site or stop codon. For example, in the case of flanking regions up to 1,500 nt away from an LCR, this would mean the flanking region from an LCR could not be used if the LCR was less than 3,000 nt away from the nearest LCR or twice the length of the flanking region. The termini of exons were not taken into account, as the DNA transcript was considered as a whole. Because LCRs are often located near the termini of proteins (Huntley and Clark 2007), there were many LCRs for which only the 5′ or 3′ flanking region could be used, as the flanking region on the opposite site of the LCR was interrupted (see supplementary fig. S1, Supplementary Material online, for distribution of maximum flanking region length and see supplementary table S1 and S2, Supplementary Material online). As well, CodeML (Yang 2007) requires that sequences being compared do not contain gaps, so flanking regions could only be used if sequences from all five primate species were present. This resulted in 1,385 upstream flanking regions and 1,399 downstream flanking regions of at least 300 nt in length from 1,136 genes; however, the number of gaps in the vicinity of LCRs lowered the number of available examples surrounding the LCR. The same subset of flanking regions was used for both the primate sequences and sequences from the 1000 Genomes Project.

**Fig. 1.—:**
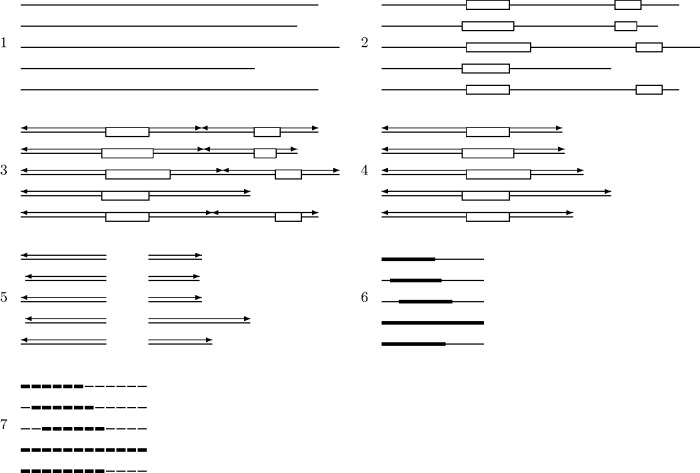
Data workflow: (1) Homologous proteins found for five primate species; (2) LCRs identified using Seg; (3) Maximum length of flanking sequences determined by protein termini and midpoints between two LCRs; (4) Flanking regions filtered for examples with homologous sequences from all five species. Because the second LCR in this example is present in only four species, its flanking regions are not used; (5) The 3′ and 5′ flanking sequences are considered separately so that if all five homologous sequences are not available, the other can still be used; (6, continuing with the 3′ sequence) The upstream and downstream flanking regions are aligned separately; gaps are represented with thin lines; (7) Alignments of individual codons are used to find the number of substitutions at each site for each flanking region with CodeML. Codons with gaps (in this case codons 1, 2, and 7–12) are not usable and are not considered when calculating the average number of substitutions per site. The number of substitutions was found for all usable sites of all genes found to contain LCRs, and the average across all usable sites was found for all positions relative to the LCR (i.e., codon 1, codon 2, etc.).

CodeML (Yang 2007) was used to estimate the number of substitutions for each codon of the flanking regions, using model 2 (two or more *d*_N_/*d*_S_ ratios for branches) for both the primate and 1000 Genomes data. As an accurate phylogenetic tree could not be provided for data from the 1000 Genomes Project, pairwise comparisons between all individuals were used instead. The mean of these pairwise comparisons was found for each site. The average number of substitutions was computed for all usable codons adjacent to the LCR (fig. 1). Pearson’s product-moment correlation (R Development Core Team 2011) was used to test for a relationship between the average number of substitutions and the distance from the LCR. To ensure that substitutions detected were not the result of misalignments, the relationship between the number of substitutions and distance from the LCR was also calculated only for flanking regions that were likely to be accurately aligned (such as flanking regions that contained no gaps when aligned).

To test whether or not selection was occurring, CodeML was used to estimate *d*_N_/*d*_S_, or ω, for each codon in flanking regions. An expected distribution of *d*_N_/*d*_S_ ratios was found for each number of substitutions by simulating codon sequences under neutral selection using evolver (part of the PAML package; Yang 2007). The *d*_N_/*d*_S_ ratios were found for each of these sets of codons and used to determine whether estimated *d*_N_/*d*_S_ ratios of the codons from flanking regions were expected under neutral selection or indicated significant evidence of positive or negative selection. Although there were a larger number of aligned codons in proximity to the LCR, this should not affect the significance of *d*_N_/*d*_S_, as the sample size used when calculating the *d*_N_/*d*_S_ ratio and generating expected distributions was fixed to five. The proportion of genes that had evidence of positive selection (i.e., a significant *d*_N_/*d*_S_ ratio greater than 1) or negative selection (i.e., a significant *d*_N_/*d*_S_ ratio less than 1) was found for each site (Yang and Bielawski 2000). The cutoff for significance, found using the expected distribution of *d*_N_/*d*_S_ values, varied depending on the number of substitutions in the aligned codon sequences. The average proportion of genes that had significant evidence of positive or negative selection was found for each aligned codon from the flanking regions, and the data were searched for a relationship between distance from the LCR and proportion of genes undergoing positive and negative selection.

As CodeML is only capable of analyzing substitutions, but not indels, any site with a gap in the alignment was ignored when calculating the average substitution rate. To study whether LCRs are associated with indels, the number of genes in which at least one sequence had a gap was found for each nucleotide. This was expressed as a proportion, i.e., as a percentage of genes that had an indel next to the LCR, 1 bp away from the LCR, 2 bp away from the LCR, etc. This data was tested for a correlation between average number of gaps per site and distance from the LCR.

To further explore the possible effects of selection, the value of Tajima’s *D* (Tajima 1989) was calculated for each codon in the flanking regions of the data from the 1000 Genomes Project. To determine the significance of each *D* value, the program ms (Hudson 2002) was used to generate a distribution of expected Tajima’s *D* values for neutrally evolving fragments with the same length and number of polymorphic sites as each codon. The Benjamini–Hochberg procedure (Benjamini and Hochberg 1995) was used to control for the false discovery rate, and the proportion of genes with evidence for positive or negative selection was found for each site.

## Results

**Fig. 2.—:**
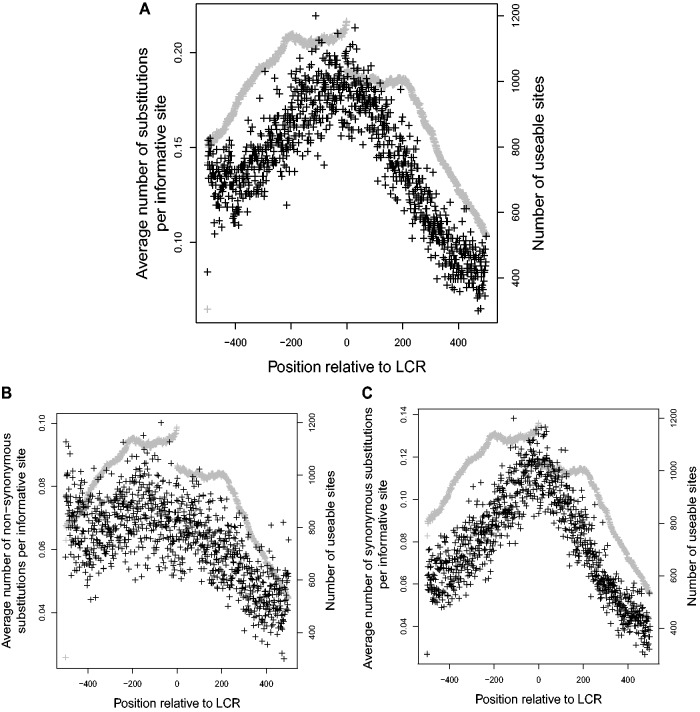
Effect of distance from LCR on average number of (*A*) total substitutions, (*B*) nonsynonymous substitutions, and (*C*) synonymous substitutions at each codon in five primate species. Gray points indicate *N*, the number of genes that could provide information and were free of gaps at each site. Negative values are upstream of the LCR.

LCR-encoding sequences were found to have a profound effect on their flanking regions. Several characteristics of the protein-coding DNA surrounding LCRs appeared to be directly affected by their distance from the nearest LCR. A strong, statistically significant negative correlation was seen between the average number of substitutions and distance from the LCR (correlation coefficient 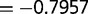, *P* value 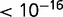, see fig. 2*A*). Although misalignments due to indels may be more likely in the vicinity of LCRs, this does not appear to be a factor, because the relationship between substitution and distance to LCR was only slightly weaker (−0.7432, *P* value 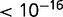) when only flanking regions that had no alignment gaps were used. As well, both synonymous and nonsynonymous substitution rates had a significant relationship to distance from the LCR, although the relationship between synonymous substitution rate and distance was stronger than the relationship between nonsynonymous substitution rate and distance (–0.8748 and –0.3787, respectively, *P* value 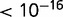 for both, see fig. 2*B* and *C*).

**Fig. 3.—:**
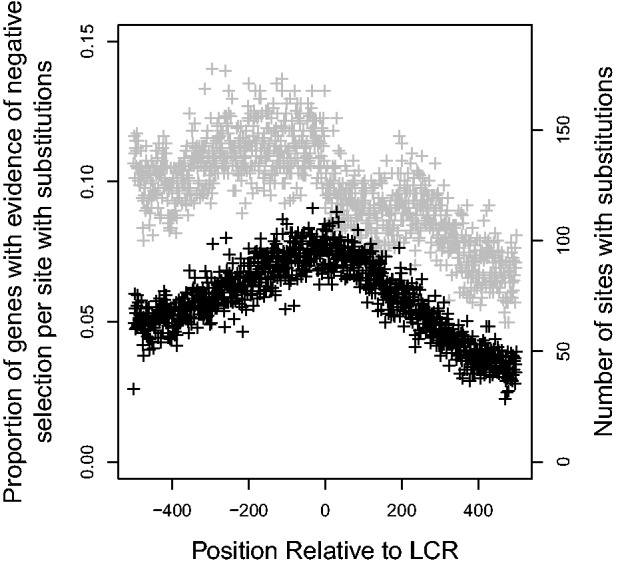
Effect of distance from LCR on proportion of flanking regions that had evidence for negative selection (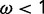). Gray points indicate the number of genes that had substitutions and could be used to calculate *d*_N_/*d*_S_. Negative values are upstream of the LCR.

**Fig. 4.—:**
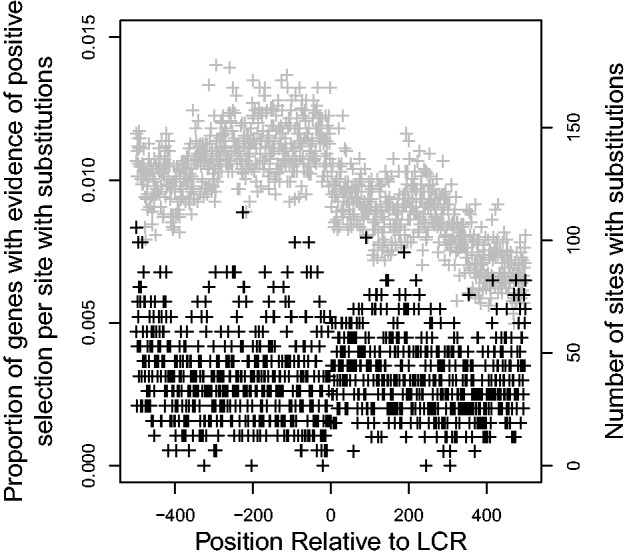
Effect of distance from LCR on proportion of flanking regions that had evidence for positive selection (ω > 1). Gray points indicate the number of genes that had substitutions and could be used to calculate *d*_N_/*d*_S_. Negative values are upstream of the LCR.

CodeML was also used for each site to estimate the *d*_N_/*d*_S_ ratio to predict the nature of selective forces acting on the LCR-flanking sequences. If *d*_N_/*d*_S_ was greater than 1, there is evidence that positive selection is occurring, while negative selection is more likely if *d*_N_/*d*_S_ is less than 1. Using the expected distributions of *d*_N_/*d*_S_ for neutrally evolving codon alignments generated using evolver to identify only significant results and ignoring sites for which *d*_N_/*d*_S_ could not be estimated (i.e., sites with gaps in the alignment or no substitutions), a strong, negative correlation was found between distance from the LCR and proportion of genes that had evidence for negative selection at each site (Pearson’s product-moment correlation, correlation coefficient 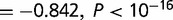, fig. 3). Like the findings of Huntley and Clark (2007), there was also a negative (although, in this case, nonsignificant) correlation between distance from the LCR and the proportion of genes that had evidence for positive selection at each site (Pearson’s product-moment correlation, correlation coefficient 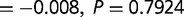, fig. 4).

**Fig. 5.—:**
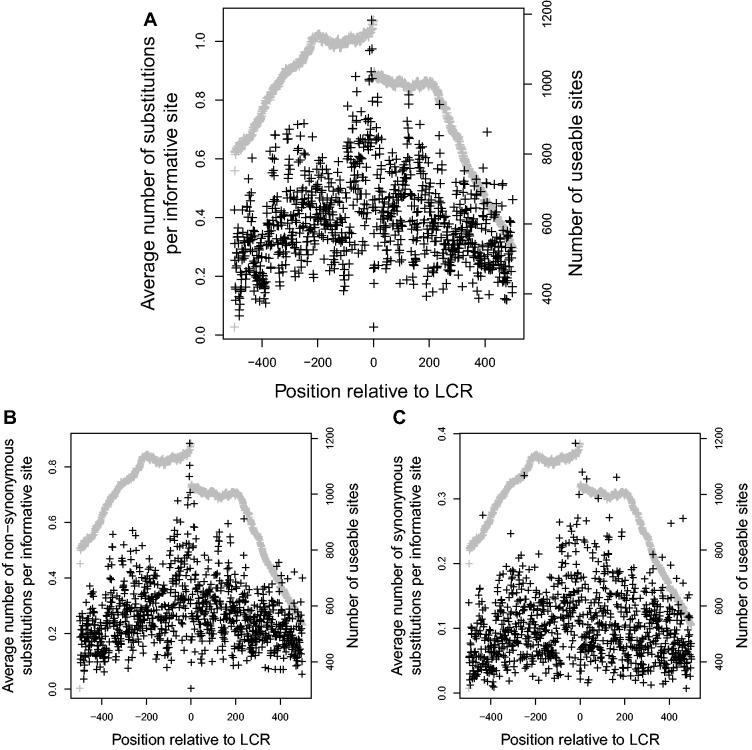
Effect of distance from LCR on average number of (*A*) total substitutions, (*B*) nonsynonymous substitutions and (*C*) synonymous substitutions at each codon in humans. Gray points indicate *N*, the number of genes that could provide information and were free of gaps at each site. Negative values are upstream of the LCR.

Using corresponding flanking regions from the 1000 Genome Project, a relationship similar to the one seen between primate sequences was found. The average number of substitutions per site had a significant negative correlation with distance from the LCR (correlation coefficient 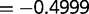, *P* value 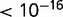, fig. 5*A*). This relationship was consistently seen, even when the number of substitutions was separated into synonymous and nonsynonymous substitutions, although the correlation between distance and nonsynonymous substitutions was slightly stronger than that for synonymous substitutions (correlation coefficients of −0.452 and −0.354, respectively, both with *P* values 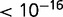, figs. 5*B* and *C*). Using the SNP calls from the Broad Institute, distance from the LCR was found to have a weak but positive relationship with the frequency of derived SNPs (Spearman’s 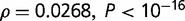). This substantiates the high substitution rate in the vicinity of LCRs, as SNPs that have lower frequencies (i.e., are more recent) are more likely to be found near LCRs.

**Fig. 6.—:**
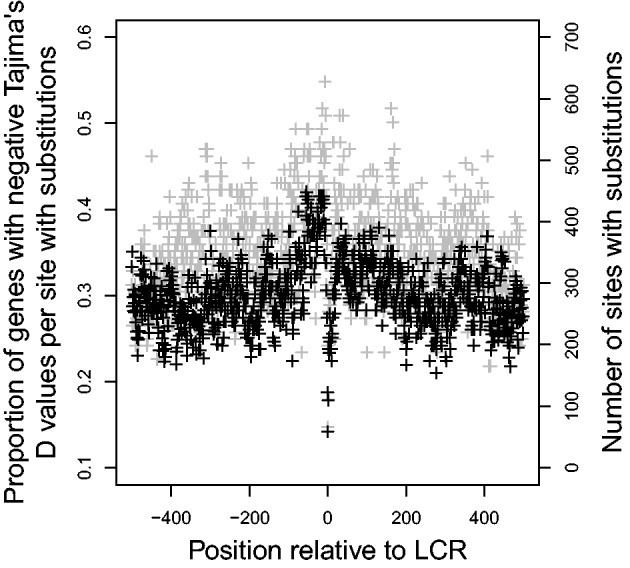
Effect of distance from the LCR on proportion of genes that had negative values for Tajima’s *D* at each codon. Gray points indicate the number of genes with substitutions that could be used to calculate Tajima’s *D* at each site. Negative values are upstream of the LCR.

As a measure of potential negative selection, we searched for negative values of Tajima’s *D*. These calculations were done for each individual codon, and the distance of the codon from the nearest LCR was recorded. Using only values of Tajima’s *D* that were found to be significant, a negative correlation was found between distance from the LCR and proportion of genes with negative values of Tajima’s *D* at each codon (correlation coefficient 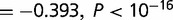, fig. 6). As a negative value for Tajima’s *D* can also indicate a bottleneck, a high number of negative values of Tajima’s *D* at any distance from an LCR can be expected between human sequences; however, the presence of an LCR clearly increases the proportion of sites in accord with more negative selection. There was also a negative relationship between distance from the LCR and proportion of genes with positive values of Tajima’s *D* at each codon, but this correlation was extremely weak and nonsignificant (correlation coefficient = −0.010, *P* = 0.7545).

**Fig. 7.—:**
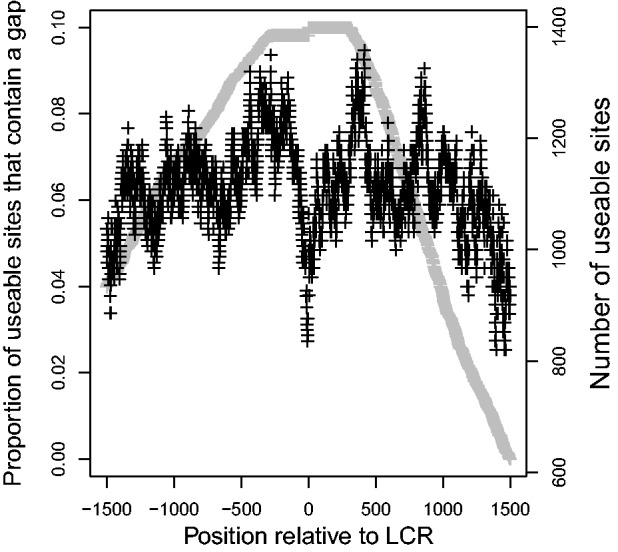
Effect of distance from the LCR on average proportion of genes with gaps in the alignment at each nucleotide in five primate species. Gray points indicate *N*, the number of genes that could provide information on each site. Negative values are upstream of the LCR.

The proportion of genes with a gap appearing in the aligned flanking regions was found and compared with distance from the LCR. There was a strong, negative relationship between distance from the LCR and number of gaps (correlation coefficient = −0.244, *P* 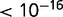, fig. 7).

The nucleotide composition of the protein-coding sequences of these genes was also investigated. The correlations between nucleotide composition in LCRs and their flanking regions were compared with the correlations between nucleotide composition in the flanking regions and the entire gene. Although there were significant correlations for all nucleotides between LCRs and flanking regions, the relationships between the flanking regions and genes were much stronger (table 2).

**Table 2:** Correlation between Nucleotide Representation in LCRs and Their Associated Flanking Regions, Compared with the Correlation between Flanking Regions and the Rest of the Protein

Nucleotide	Correlation between Flanking Region and LCR	Correlation between Flanking Region and Protein
A	0.5070	0.6635
C	0.4152	0.6206
G	0.3343	0.5415
T	0.2726	0.5395

Note.— All correlations were significant (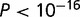).

A search of the Gene Ontology database (Gene Ontology Consortium 2000) revealed no major trends. The functions seen most often are extremely common and likely to be shared among many unrelated genes.

## Discussion

Although the evolution of repetitive regions has been studied for many years, the study of repetitive regions within coding regions is comparatively more recent. In noncoding regions, repetitive regions are known to evolve far more rapidly than nonrepetitive sequences due to both base substitutions and indels generated by slippage (Levinson and Gutman 1987). Indeed, microsatellites are described as one of the most variable types of DNA sequence in the genome (Ellegren 2004). Microsatellites have many mutational biases and are known to lead to multiple mutational events (Drake 2007; Schrider et al. 2011) and to mutational nonindependence (Amos 2010a). Microsatellites have also been shown to increase the frequency of mutations in the surrounding nucleotide sequences (Amos 2010c, 2010b; Varela and Amos 2010).

In addition to intergenic regions, repetitive sequences are also present in protein coding regions (Golding 1999; Marcotte et al. 1999). They too evolve rapidly (Huntley and Golding 2002; Alba and Guigo 2004; Huntley and Clark 2007; Brown et al. 2010; Haerty and Golding 2010) and have unusual mutation properties including the suggestion that the rate of substitutions are elevated in the surrounding nonrepetitive coding sequence (Huntley and Clark 2007; Haerty and Golding 2011). The few attempts made to assess the evolution of the genetic variation in the surrounding sequences of repeats reported an absence of selection on these sites (Jovelin and Cutter 2013). However, despite the fact that previous studies found repeated sequences to be preferentially located within coding regions under relatively low selective constraints (Romero et al. 2001; Haerty and Golding 2010), purifying selection is still expected to act on those SNPs arising in the vicinity of these LCRs because of the protein-coding sequence context. The extensive data now available for human and other primate genomes allows a more precise evaluation of the mutational potential of LCRs and the evolution of their flanking sequences.

In coding regions surrounding LCR sequences, we identified a relationship between the presence of LCRs and increased local substitution rates for humans and related primates. Not only is the correlation statistically significant, but the average number of substitutions is higher in sequences near LCRs and decreases rapidly as distance from the LCR increases. This demonstrates that the relationship between proximity to LCRs and substitution rate seen in previous studies, *E. coli*, yeast (McDonald et al. 2011), *Drosophila* (Huntley and Clark 2007; McDonald et al. 2011), *Caenorhabditis* (Jovelin and Cutter 2013), and *Plasmodium* (Haerty and Golding 2011), is also present in primates. It is also consistent with previous findings showing an increased number of mismatches and SNPs in the vicinity of microsatellites (Vowles and Amos 2004; Siddle et al. 2011), suggesting that the repetitiveness of these sequences is key to their increased mutation rate. This relationship between LCR distance and substitution is also apparent for substitutions segregating within humans, as shown using the data from the 1000 Genomes Project. Along with the substitution rate, the number of gaps in the aligned sequences appears to be greater in the vicinity of LCRs. From these results, it is clear that the frequency of both point mutations and indels increases in DNA immediately surrounding LCRs in primates.

The specific subset of flanking regions used in this analysis (1,385 upstream and 1,399 downstream flanking regions) should accurately represent the genome-wide effect of protein-coding LCRs on adjacent DNA. The criteria by which sequences were eliminated from this analysis was the length of flanking regions, determined by the distance from LCRs to the protein termini, or, in examples where there were multiple LCRs per gene, by the distance between LCRs. No analyses have been performed to determine whether there is a relationship between the protein function and the location or number of LCRs, but no trends were found in the GO functional classification of the proteins used in this study. Although flanking regions from only a fraction of LCRs originally detected were used, the diverse array of functions suggests that this is a representative sample of LCR-containing genes.

Although the flanking regions clearly show a relationship between distance from the LCR and substitution rate, they also show asymmetry in that relationship. The substitution rate is higher on the 5′ side of the LCR-encoding sequence. Although the nature of the mutational pressure affecting DNA adjacent to LCRs is still uncertain, the skewness of the substitution rates could implicate a transcription-related mechanism. Most of the LCRs used here were expressed in germline cells (expression patterns found using the Human Protein Atlas (Uhlen et al. 2012); 1,108 upstream and 1,119 downstream flanking regions were expressed in the germline and 277 upstream and 280 downstream flanking regions were not expressed in the germline), but the relationship between distance from the LCR and substitution rate remains skewed even for cells not expressed in the germline (supplementary figs. S2 and S3, Supplementary Material online). Polak et al. (2010) found a substitution transcriptional bias in DNA, where regions just downstream of the transcription start site are more prone to substitutions that lead to strong bonds (i.e., more CG base pairs, with three hydrogen-bonds, than TA pairs); an excess of C to T substitutions over G to A was also found near the transcription start site in some genes. Although a bias in substitution rate was not found, the difference in substitutions based on proximity to the transcription start site could be related to the asymmetry in the number of substitutions seen around LCRs.

Hence, our observation of a transcriptional asymmetry in the SNP distribution broadens the mechanism proposed by McDonald et al. (2011) to explain the mutagenic effect of repeats. In their model, McDonald et al. (2011) associated the increased mutation rate within the flanking sequences of microsatellites to the recruitment of error-prone DNA-polymerases at stalled replication forks. The presence of highly repetitive sequence within protein-coding sequences is likely to also stall the RNA-polymerase (Parsons et al. 1998), leading to DNA damage in the transcribed strand followed by transcription coupled repair or nucleotide excision repair.

A possibly interesting result is the distinct dip adjacent to the LCR in both the proportion of genes with negative values of Tajima’s *D* and the proportion of genes with gaps. The decrease in indel mutations immediately adjacent to the LCR may indicate that DNA at the borders of LCRs is important for the correct alignment of these protein-coding regions during recombination. It is however possible that this is simply due to alignment artifacts. These alternatives have not yet been distinguished.

The LCRs themselves have variations in length that can cause inaccurate alignments; a slightly more stable region surrounding LCRs could mitigate the effects of this. However, there was also a drop in the proportion of genes with negative values of Tajima’s *D* in this area, suggesting that the DNA immediately adjacent to the LCRs is less conserved. This could be a result of the misidentification of the exact borders of the LCR, as these few sites might be under different selection pressures if they were part of the LCR itself.

Jovelin and Cutter (2013) applied a similar procedure for the SNPs in the vicinity of indels within up to 217 sequences surrounding miRNA genes in *Caenorhabditis*. In contrast to our results, no significant evidence for a skew in the distribution of allele frequency was observed in nematodes. The differences between the studies may be a consequence of the fact that we selected LCRs within protein-coding sequences and also the increased power we gained by interrogating SNPs across the whole human genome.

There are several possible causes for the high substitution rate in LCRs that have been previously studied (Levinson and Gutman 1987; Moore et al. 1999; Amos et al. 2008), but the reason why this effect should extend into flanking regions has not yet been determined. One of the major factors believed to be affecting the mutation rate in LCRs is replication slippage, which causes the LCR to expand or contract by one or more repeated units during replication. Mutations resulting in LCRs of varying lengths could affect the surrounding DNA, as misalignments could make DNA repairs in flanking regions more error prone. The increase in the number of SNPs surrounding mononucleotide repeats found by Siddle et al. (2011) supports this, as expansion and contraction mutations are more likely in repetitive sequences and both are potential causes of misalignment. Another less-explored possibility is unequal recombination. Due to the repetitive nature of LCRs, alignment during crossover events is sometimes inaccurate. This can lead to unequal DNA sequences being exchanged between two strands of DNA. These recombination events can also incorporate DNA adjacent to the LCR, which would explain the high substitution rate of LCR-flanking regions. As well, more gaps would be expected if unequal recombination were likely, and a strong correlation was seen between distance from the LCR and the proportion of genes with gaps seen in the alignment.

There is also an alternative possibility that these flanking regions were previously part of the LCR, created through the LCR’s expansion. Later, point mutations, which can increase the complexity of an LCR sometimes to the point of causing LCR death (Radó-Trilla and Albà 2012), might then change the sequence enough to obscure its origin within an LCR. The high substitution rate in regions flanking LCRs would then be a result of the LCR’s expansion and high substitution rate. As well, the natural propensity for expansion and contraction of LCRs would then still be expected to have an effect on regions on the verge of gaining complexity through point mutations, leading to the relationship between the number of gaps seen and distance from the LCR. If this were the case, however, flanking regions would be expected to have a nucleotide composition more similar to their associated LCRs than to the rest of the protein, which was not seen.

It is also possible that the increased number of substitutions surrounding LCRs is due to relaxed selection allowing more substitutions. The data from CodeML directly contradict this, however, as many sites in flanking regions had *d*_N_/*d*_S_ ratios that indicated significant evidence for negative selection. The flanking regions of LCRs may be subject to more synonymous and nonsynonymous substitutions, but synonymous substitutions are more prevalent than nonsynonymous. As the probability of seeing negative selection significantly increases in proximity to the LCR, it appears that the region surrounding the LCR is under strong selection pressure favoring synonymous substitutions. This intolerance for changes to the protein sequence indicates functional importance in the regions around LCRs.

Using Tajima’s *D* to test what type of selection was likely to be occurring within data from the 1000 Genomes Project data, negative values were represented far more often than positive, especially in proximity to LCRs, likely indicating purifying selection. As a negative Tajima’s *D* can also indicate recent population expansion, some of these values could be the result of the recent evolutionary history of humans, but demographic history would not be expected to result in an apparent relationship between Tajima’s *D* and distance from an LCR. The relationship between Tajima’s *D* and distance from the LCR therefore also conforms with the relationship seen in the primate species, providing more evidence for a functional importance in flanking regions.

## Conclusions

It is clear that substitution rates are greatly increased in LCRs, and this effect extends into the flanking regions beyond the LCR. There is a strong correlation between distance from an LCR and number of changes seen between sequences. This correlation exists for both synonymous and nonsynonymous changes while a lesser correlation exists for indels. Average *d*_N_/*d*_S_ ratios indicate that the regions around LCRs may be tightly controlled, as negative selection appears to be far more common than positive selection.

## Supplementary Material

Supplementary figures S1–S3 and tables S1–S2 are available at *Genome Biology and Evolution* online (http://www.gbe.oxfordjournals.org/). 

Supplementary Data
