# Supplementary material for: Increased Substitution Rates Surrounding Low-Complexity Regions within Primate Proteins
Source: Genome Biol Evol. 2014 Feb 25;6(3):655–65. doi: 10.1093/gbe/evu042 (PMC3971593; doi:10.1093/gbe/evu042)
Supplement: Supplementary Data [file supp_6_3_655__index.html]

Increased substitution rates surrounding low-complexity regions within primate proteins — Increased Substitution Rates Surrounding Low-Complexity Regions within Primate Proteins — Supplementary Data 

# Increased Substitution Rates Surrounding Low-Complexity Regions within Primate Proteins

## Supplementary Data

file

**Files in this Data Supplement:**

- Supplementary Data - zip file
